# The Commensal Microbiota Enhances ADP-Triggered Integrin α_IIb_β_3_ Activation and von Willebrand Factor-Mediated Platelet Deposition to Type I Collagen

**DOI:** 10.3390/ijms21197171

**Published:** 2020-09-28

**Authors:** Klytaimnistra Kiouptsi, Sven Jäckel, Eivor Wilms, Giulia Pontarollo, Jana Winterstein, Cornelia Karwot, Kathrin Groß, Kerstin Jurk, Christoph Reinhardt

**Affiliations:** 1 Center for Thrombosis and Hemostasis (CTH), University Medical Center of the Johannes Gutenberg- University of Mainz, Langenbeckstrasse 1, 55131 Mainz, Germany; Klytaimnistra.Kiouptsi@unimedizin-mainz.de (K.K.); svenjaeckel@gmx.de (S.J.); eivorwilms@googlemail.com (E.W.); Giulia.Pontarollo@unimedizin-mainz.de (G.P.); janawinterstein@web.de (J.W.); cornelia.karwot@unimedizin-mainz.de (C.K.); Kathrin.Gross@unimedizin-mainz.de (K.G.); kerstin.jurk@uni-mainz.de (K.J.); 2 German Center for Cardiovascular Research (DZHK), Partner Site RheinMain, 55131 Mainz, Germany

**Keywords:** microbiota, germ-free, von Willebrand factor, Toll-like receptor-2, platelets, α_IIb_β_3_

## Abstract

The commensal microbiota is a recognized enhancer of arterial thrombus growth. While several studies have demonstrated the prothrombotic role of the gut microbiota, the molecular mechanisms promoting arterial thrombus growth are still under debate. Here, we demonstrate that germ-free (GF) mice, which from birth lack colonization with a gut microbiota, show diminished static deposition of washed platelets to type I collagen compared with their conventionally raised (CONV-R) counterparts. Flow cytometry experiments revealed that platelets from GF mice show diminished activation of the integrin α_IIb_β_3_ (glycoprotein IIbIIIa) when activated by the platelet agonist adenosine diphosphate (ADP). Furthermore, washed platelets from Toll-like receptor-2 (*Tlr2*)-deficient mice likewise showed impaired static deposition to the subendothelial matrix component type I collagen compared with wild-type (WT) controls, a process that was unaffected by GPIbα-blockade but influenced by von Willebrand factor (VWF) plasma levels. Collectively, our results indicate that microbiota-triggered steady-state activation of innate immune pathways via TLR2 enhances platelet deposition to subendothelial matrix molecules. Our results link host colonization status with the ADP-triggered activation of integrin α_IIb_β_3_, a pathway promoting platelet deposition to the growing thrombus.

## 1. Introduction

While genetic predisposition to arterial thrombosis is increasingly understood [1], the knowledge of environmental modifiers contributing to cardiovascular disease (CVD) progression and arterial thrombosis remains elusive [2]. One of the environmental factors affecting arterial thrombus growth is the commensal microbiota. The microbiota, a highly diverse ecosystem consisting of trillions of microbes colonizing our body surfaces, is established at birth [3,4]. These microbial communities interact dynamically with their host, forming a metaorganism, thus influencing host physiology [5,6,7]. In recent years, microbial sequencing studies on patient cohorts, as well as experimental work on gnotobiotic mouse models has linked the gut microbiota to the development of CVD and arterial thrombosis [8,9,10].

Numerous metabolic pathways are influenced by the microbiota, dependent on nutrition [11,12]. However, in addition to signaling-active microbiota-derived metabolites that are largely determined by the availability of nutrients, the microbiota provides a broad variety of microbial-associated molecular patterns (MAMPs) that steadily trigger the basal activation of pattern recognition receptors, such as Toll-like receptors (TLRs) [13,14]. These pro-inflammatory signals are not restricted to the surface of the intestinal epithelium, but remain active and can be detected at varying levels in the bloodstream, dependent on gut barrier function [15,16,17]. Remarkably, MAMPs derived from the gut microbiota can signal to distant vascular beds, influencing both endothelial cell function and platelet responses [18,19,20]. In the vasculature, remote signaling of microbial patterns is mediated by TLRs and nucleotide-binding oligomerization domain (NOD)-like receptors [15,21], impacting on hematopoiesis but also affecting the development of vascular disease states such as atherosclerosis, ischemia-reperfusion injury, and arterial thrombosis [22,23,24].

Recent research on the vascular physiology of germ-free (GF) mouse models has linked colonization with commensal microbiota to CVD development and its most severe complication, arterial thrombosis [2,9,25,26,27,28]. In spite of the recognized role of the microbiota in enhancing platelet adhesion to subendothelial matrix components and arterial thrombus formation [9,29], so far little is known on the precise molecular mechanisms that are influenced by the presence of gut microbiota and that support arterial thrombus growth. Primary adhesion of platelets to subendothelial collagen is mediated via the interaction of the GPIb-V-IX receptor complex with exposed von Willebrand factor (VWF). Integrin α_2_β_1_ and subsequent GPVI signaling stabilizes the interaction of platelets with collagen type I at static conditions [30,31]. Thereafter, thrombus growth is predominantly supported by the abundantly expressed platelet integrin α_IIb_β_3_ (the GPIIbIIIa receptor) after its activation [32]. In addition to the recognized role in the formation of inter-platelet fibrinogen-bridges, the platelet integrin α_IIb_β_3_ can also interact with VWF to mediate stable adhesion to the subendothelium through binding to the VWF RGD (Arg-Gly-Asp)-motif in a Ca^2+^-dependent manner [33]. This Arg-Gly-Asp (RGD) binding motif is situated in the C-terminal region of VWF, mediating its binding to the activated platelet integrin α_IIb_β_3_ [34]. In static adhesion experiments, it was demonstrated that platelets bind to immobilized VWF and fibrinogen [35]. While inhibition of the platelet-activating pathways with prostaglandin E1 (PGE1) and forskolin prevented static platelet deposition to VWF, it did not block deposition to fibrinogen. Therefore, it is clear that pre-activation is an essential requirement for the integrin α_IIb_β_3_-mediated VWF-platelet interaction under static conditions.

Our recent investigations on germ-free (GF) mouse models have revealed a microbiota-triggered Toll-like receptor-2 (TLR2)-mediated pathway that promotes hepatic VWF synthesis, thus supporting integrin α_IIb_β_3_-dependent platelet deposition onto laminin matrices [19]. Furthermore, we demonstrated in the hyperlipidemic low-density lipoprotein-receptor (*Ldlr*)-deficient mouse model that the platelets of GF *Ldlr*-deficient mice, which were kept on an autoclaved standard laboratory diet, had reduced activation of the integrin α_IIb_β_3_ when adhering to a collagen type III matrix relative to conventionally raised (CONV-R) *Ldlr*-deficient controls. These results, obtained ex vivo in a standardized flow chamber model, suggest a diminished adhesion-dependent activation of GF mouse platelets [27]. This observation is in line with previous work indicating that GF mice have increased bleeding times associated with a reduced collagen-stimulated platelet activation [29]. However, it is unresolved whether the commensal microbiota affects the amplifying pathway triggered upon tethering of platelets to subendothelial matrix, e.g., via the ADP-stimulated platelet activation.

To gain mechanistic insights on the microbiota’s role in promoting platelet deposition to subendothelial matrix components, we here studied GF and *Tlr2*-global-knockout mouse models. Comparing GF with CONV-R mice as well as WT with *Tlr2*-deficient mice, we analyzed static platelet deposition to type I collagen coatings and ADP-induced activation of α_IIb_β_3_. We found that the platelets from GF mice show reduced deposition to type I collagen coatings, paralleled by impaired ADP-stimulated activation of the integrin α_IIb_β_3_. Similar to the adhesion of GF mouse platelets, platelets from *Tlr2*-deficient mice that lack innate immune signaling via TLR2 also showed reduced deposition to type I collagen. The observed platelet deposition phenotype was strongly dependent on the plasma source as pre-incubation with WT plasma rescued diminished platelet deposition to type I collagen.

## 2. Results

### 2.1. Platelets from Germ-Free Mice Show Reduced Deposition to Type I Collagen and Diminished ADP-Induced α_IIb_β_3_ Integrin Activation

**Figure 1:**
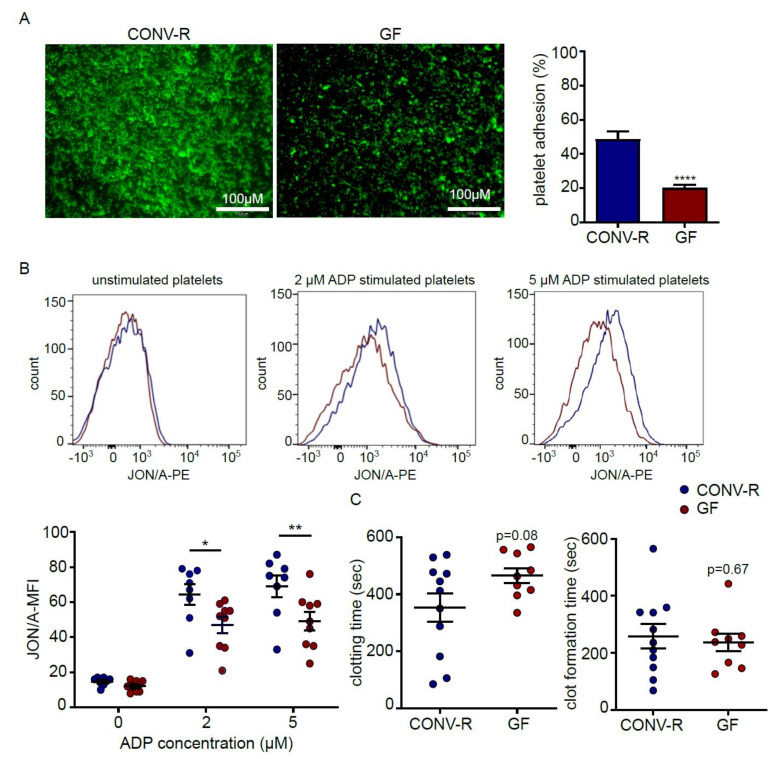
Reduced deposition of germ-free (GF) mouse platelets to type I collagen coatings is paralleled by diminished ADP-triggered integrin α_IIb_β_3_ activation. (**A**) Representative images and bar graph of platelet deposition from GF (red) and conventionally raised (CONV-R) (blue) mice on type I collagen-coated slides (*n* = 13) (**B**) Flow cytometry analysis of platelets from GF (red) vs CONV-R (blue) mice stimulated with 0, 2 or 5 μM ADP and stained with the phytoerythrin (PE)-conjugated JON/A antibody, recognizing activated integrin α_IIb_β_3_; representative histograms and MFI (mean fluorescence intensity) quantifications (*n* = 9, 8). (**C**) Comparative analysis of clotting time (CT) and clot formation time (CFT) in collagen-stimulated whole blood (8 µg/mL) from GF and CONV-R mice (*n* = 9, 11). All data are expressed as means ± SEM. Statistical comparisons were performed using the Student’s *t*-test and two-way ANOVA. * *p* < 0.05, ** *p* < 0.01, **** *p* < 0.0001.

Type I collagen is a major component of the subendothelial matrix that is exposed after vascular injury (e.g., during atherosclerotic plaque rupture). Here we analyzed the deposition of isolated washed platelets from CONV-R and GF mice onto type I collagen coatings ex vivo. In line with our previous work, demonstrating reduced platelet deposition to the subendothelial matrix constituent laminin [19], washed platelets from GF mice showed an approximate 50% reduction in platelet deposition onto type I collagen matrices (Figure 1A). Hence, platelets from GF mice are characterized by an impaired deposition to subendothelial matrix components [19,29].

Because thrombus growth is primarily dependent on intact integrin α_IIb_β_3_ function [32], we hypothesized that the observed reduced deposition of GF mouse platelets to type I collagen coatings could be due to impaired adhesion-induced integrin α_IIb_β_3_ activation that, in addition to other agonists, is efficiently promoted by the platelet agonist ADP, secreted from platelet-dense granules. Therefore, we stimulated isolated platelets from GF and CONV-R mice with different ADP concentrations. Flow cytometry analyses revealed that upon ADP stimulation, platelets isolated from GF mice were less reactive in terms of integrin α_IIb_β_3_ activation (Figure 1B).

To test whether differences in the collagen-induced activation of the clotting system in whole blood could be involved in the diminished deposition of GF mouse platelets, we analyzed citrate-anticoagulated whole blood that was stimulated with type I collagen by thrombelastometry. With this method, we found a trend towards a prolonged clotting time (CT) in GF type I collagen-stimulated whole blood, indicating an impaired coagulation response (Figure 1C, left panel). In contrast, clot formation time (CFT) was similar in GF and CONV-R citrate-anticoagulated mouse blood (Figure 1C, right panel), indicating that platelet function between GF and CONV-R mice was comparable in this ex-vivo assay.

Collectively, our results identified a diminished ADP-induced activation of the platelet integrin α_IIb_β_3_ at GF housing conditions, a major platelet signaling loop that could explain the reduced static platelet deposition to type I collagen, observed with GF mouse platelets.

### 2.2. Deficiency in TLR2 Results in Impaired VWF-Mediated Platelet Deposition to Type I Collagen

**Figure 2:**
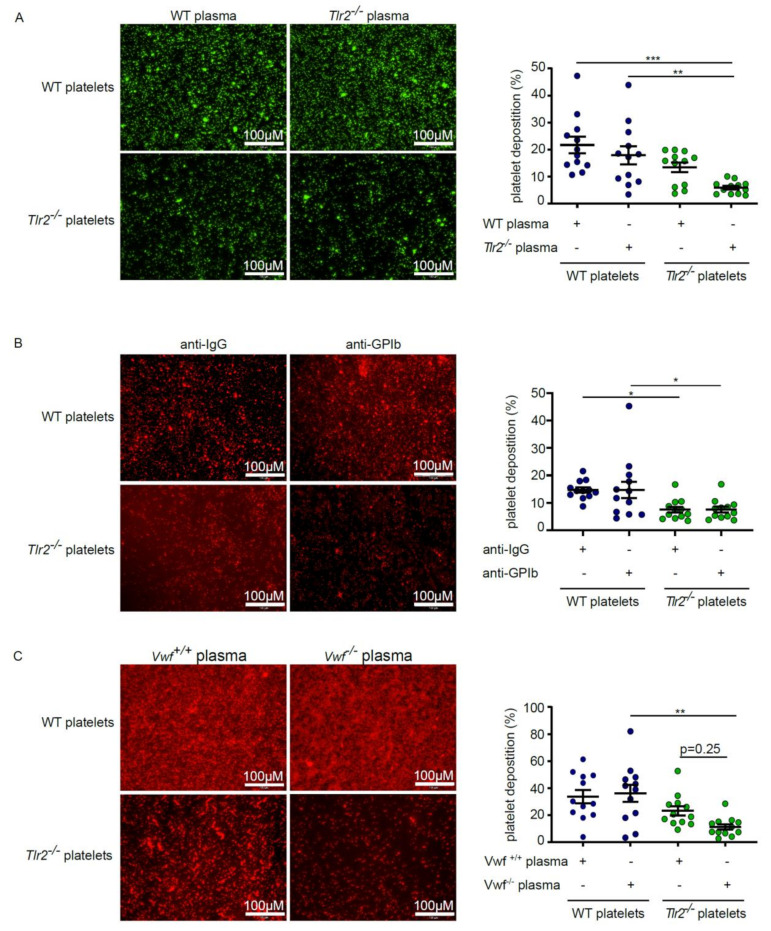
Washed platelets from *Tlr2*-deficient mice (*Tlr2^-/-^*) show a reduced von Willebrand factor (VWF)-dependent deposition onto type I collagen matrix. (**A**) Representative images and dot plot of platelet deposition (DCF-stained) from wild-type (WT) platelets incubated with plasma isolated from WT (blue) or *Tlr2^−/−^* (green) mice, and similarly *Tlr2^−/−^* platelets incubated with either plasma isolated from WT or *Tlr2^−/−^* mice on collagen coated slides (*n* = 12) (**B**) Blockade of GPIb-function in the static deposition model of isolated WT (blue) and *Tlr2^−/−^* (green) platelets (rhodamin B-stained) to type I collagen coatings (*n* = 12) and isotype control. (**C**) Representative images and dot plot of platelet deposition (rhodamin B-steined) from WT platelets (blue) incubated with plasma isolated from WT (*Vwf^+/+^*) or *Vwf^−/−^* mice and similarly *Tlr2^−/−^* platelets (green) incubated with either plasma isolated from WT (*Vwf^+/+^*) or *Vwf^−/−^* mice on collagen coated slides (*n* = 12). All data were expressed as means ± SEM. Statistical comparisons were performed using one-way ANOVA. * *p* < 0.05, ** *p* < 0.01, *** *p* < 0.001.

Toll-like receptors (TLRs) are pattern-recognition receptors involved in innate immunity that, after recognition of highly conserved molecular determinants, promote vascular thrombosis [36]. In previous work, we showed that microbiota-stimulated TLR2 signaling on the hepatic endothelium supports VWF-mediated platelet deposition to laminin, dependent on platelet integrin α_IIb_β_3_ binding [19]. To test whether plasma from *Tlr2*-deficient mice likewise suppresses platelet deposition to type I collagen, we incubated platelets from *Tlr2*-deficient mice and wild type (WT) controls, resuspended in either WT or *Tlr2*-deficient plasma on type I collagen coatings. Similar to platelet deposition to laminin coatings [19], WT platelets showed a robust deposition, no matter if they were pre-incubated with WT plasma or plasma from *Tlr2*-deficient mice (Figure 2A). In contrast, *Tlr2*-deficient platelets displayed defective platelet deposition to type I collagen, which was partially rescued when pre-incubated with the plasma from WT mice (Figure 2A). Our results confirm that integrin α_IIb_β_3_ activation is a major factor in static platelet deposition [19], but they also imply a plasma-dependent role for TLR2 signaling in modulating pro-adhesive platelet function.

To exclude a possible role of GPIbα in this model of static platelet deposition to type I collagen coatings, we blocked GPIbα in the washed platelet preparations with a functional inhibitory antibody (Figure 2B). Importantly, *Tlr2*-deficient platelets treated with isotype control antibody consistently showed a reduced percentage of platelet-covered area relative to WT controls, confirming the platelet deposition defect of the *Tlr2*-deficient mouse line. Interestingly, pre-incubation with functional inhibitory anti-GPIbα did neither reduce platelet deposition of WT platelets nor did it further diminish the platelet-covered area in the *Tlr2*-deficient group (Figure 2B). In addition, we performed flow cytometry on the platelet surface expression of other collagen receptors, such as GPVI, the integrin α_IIb_-subunit, GPIX, and the integrin α_2_-subunit, but did not note changes (Figure S1). Therefore, similar to deposition to laminin [19], the defective deposition of *Tlr2*-deficient platelets to type I collagen can likely be attributed to diminished integrin α_IIb_β_3_ function.

As we have previously demonstrated that the presence of microbiota regulates murine plasma VWF levels, leading to increased integrin α_IIb_β_3_-mediated platelet adhesion and thrombus growth in the carotid artery ligation model [19], we next studied the influence of plasma from *Vwf*-deficient (*Vwf^−/−^*) mice on platelet deposition to collagen coatings. Surprisingly, pre-incubation of washed WT platelets with either *Vwf*-deficient mouse plasma or plasma from WT controls did not lead to changed platelet deposition to type I collagen (Figure 2C). However, incubation of washed *Tlr2*-deficient platelets with plasma from *Vwf*-deficient mice resulted in vastly impaired platelet deposition onto type I collagen coatings, which was partially reduced by incubating the washed *Tlr2*-deficient platelets with WT-plasma (*Vwf^+/+^*) (Figure 2B). In essence, this experiment stresses that plasma VWF levels are a critical determinant of static platelet deposition to a type I collagen matrix in *Tlr2^−/−^* mice but not in WT controls.

**Figure 3:**
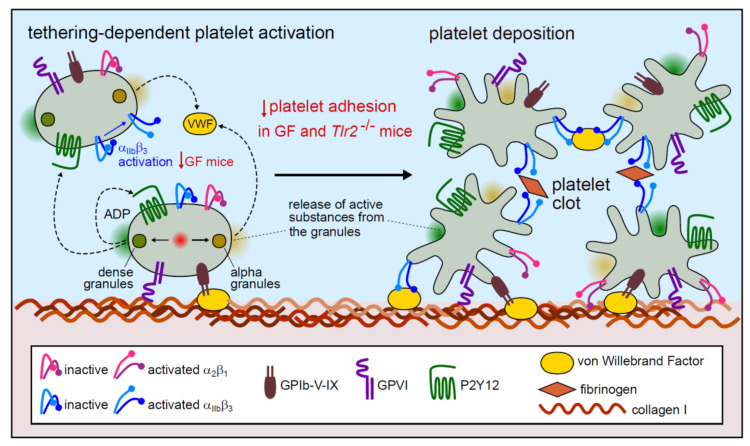
Model of the microbiota-modulation of static platelet deposition to type I collagen matrix. Primary platelet tethering to collagen is mediated by the GPIb-V-IX complex and the platelet GPVI receptor (purple). Subsequent platelet activation leads to α and dense granule secretion, the latter coupled with ADP release, resulting in integrin activation and firm platelet adhesion to collagen through the α_2_β_1_ integrin (pink) and to binding of VWF via the integrin α_IIb_β_3_ (blue). The integrin α_IIb_β_3_ also contributes to platelet aggregation by the formation of fibrinogen bridges, in both the inactive and the activated form.

Altogether, our results support the conclusion that adhesion-dependent platelet activation on a type I collagen matrix results in the pre-activation of tethering platelets and to the local exposure of ADP from platelet dense granules and VWF from platelet α-granules (Figure 3). Released ADP locally promotes the activation of integrin α_IIb_β_3_, a process that is impaired in the platelets of GF mice lacking a commensal microbiota. Diminished integrin α_IIb_β_3_ activation on the surface of GF platelets results in impaired binding to the VWF RGD-motif, which in contrast to fibrinogen binding depends on the activated form of the α_IIb_β_3_ integrin [35]. This functional difference could explain the reduced deposition of isolated GF platelets relative to CONV-R controls at static conditions.

## 3. Discussion

Our results identified the gut microbiota as an actuating variable of integrin α_IIb_β_3_ activation, which in our experimental conditions was triggered by ADP. This might at least in part explain the observed impairment in static platelet deposition of GF mouse platelets to type I collagen, a platelet deposition defect that is comparable to what we observed with platelets from *Tlr2*-deficient mice and that is determined by the exposure to varying VWF plasma levels [19]. Thus, impaired platelet integrin α_IIb_β_3_ activation in the absence of microbiota in the GF mouse model could further elucidate how commensals promote VWF-mediated platelet deposition to exposed subendothelial matrix (i.e., to collagen and laminin) [19,27].

Our results are in support of a previous study that demonstrated a tendency of prolonged tail bleeding times in GF mice, a VWF-dependent process, which the authors linked to a decreased activation of GF platelets in response to type I collagen stimulation, as indicated by reduced release of granulophysin and reduced surface exposure of P-selectin and activated integrin α_IIb_β_3_ [29]. Since this early study did not test the role of commensals in ADP-induced platelet activation but rather addressed how activated integrin α_IIb_β_3_ is critically involved in static platelet deposition [35], our data explain the observed impairment of integrin α_IIb_β_3_-mediated platelet deposition in the GF mouse model, which is a critical step in the platelet activation process [19]. Furthermore, in line with our previous study with whole blood from hyperlipidemic mice, demonstrating reduced adhesion-dependent activation of GF mouse platelets [27], and in accordance with diminished collagen-triggered platelet activation, we noted a tendency towards a prolonged clotting time in thrombelastometry experiments on the whole blood of GF mice relative to CONV-R counterparts. Hence, our results for the first time demonstrate that the ADP-triggered activation of integrin α_IIb_β_3_, an important functional signaling loop of platelets that synergizes with other platelet adhesion receptors and contributes to deposition to type I collagen matrix under various conditions, is regulated by commensal microbiota.

Thrombus growth is a concerted process that not only relies on efficient integrin α_IIb_β_3_ activation, but it critically depends on the quantity of bridging ligands in plasma that may interact with the activated integrin. In previous work, we discovered that the extent of platelet deposition to laminin matrices is related to plasmatic VWF levels [19]. Indeed, the collagen–VWF interaction is functionally important as it determines the platelet-release reaction, i.e., the secretion of granula and the exposure and activation of adhesion receptors such as integrin α_IIb_β_3_ [37,38]. Furthermore, our previous work on GF mouse models showed that plasmatic VWF levels are regulated by the presence of commensal microbiota through the sensing of microbiota-derived TLR2 agonists by the hepatic endothelium [19,21]. Here, we propose that, similar to the diminished laminin deposition of *Tlr2*-deficient platelets, the deposition of *Tlr2*-deficient platelets to type I collagen coatings is likewise reduced and can be partially rescued by pre-incubation in WT plasma containing higher levels of VWF. As expected, we found platelet deposition to type I collagen to be related to VWF plasma levels. The relevance of TLR2 in arterial thrombosis is complemented by a clinical study in systemic lupus erythematosus patients, which has identified single nucleotide polymorphisms in the *Tlr2* gene that were linked to arterial thrombosis [39].

In conclusion, our study provides new evidence for the involvement of TLR2 signaling in the regulation of pro-thrombotic platelet function and the role of the gut microbiota in tuning the sensitivity of integrin α_IIb_β_3_-activating pathways contributing to platelet deposition. Future work should address the role of the commensal microbiota in relation to other vascular TLRs and platelet receptors to define how steady-state microbiota-derived innate immune activation contributes to arterial thrombus formation.

## 4. Materials and Methods

### 4.1. Animals

C57BL/6J WT and *Tlr2^−/−^* mice on a C57BL/6J background were purchased from The Jackson Laboratory (Bar Harbor, ME, USA). *Vwf−/−* mice on a C57BL/6J background were provided by Bernhard Nieswandt. All experimental animals were 8–14 weeks old male or female mice (at least 20 g body weight) housed in the Translational Animal Research Center (TARC) of the University Medical Center Mainz under specific pathogen free (SPF) or GF conditions in EU type II cages with 2–5 cage companions with standard autoclaved lab diet and water ad libitum, 22 ± 2 °C room temperature and a 12 h light/dark cycle. All groups of mice were sex, age, and weight matched and were free of clinical symptoms. All procedures performed on mice were approved by the local committee on legislation on protection of animals (Landesuntersuchungsamt Rheinland-Pfalz, Koblenz, Germany; 23177-07/A12-1-006).

### 4.2. Blood Collection and Platelet Adhesion Under Static Conditions

Blood collection was performed as previously described [19]. Briefly, citrated whole blood was collected by intracardial puncture and centrifuged at 100× *g* for 10 min without break at room temperature. Platelet-rich plasma was supplemented with 3 mL of modified Tyrode’s buffer (137 mM NaCl, 2 mM KCl, 12 mM NaHCO_3_, 0.3 mM NaH_2_PO_4_, 5.5 mM glucose, 5 mM Hepes, 15 μΜ BSA, pH 6.5) [40] and labeled with 5-carboxyflourescein diacetate succinimidyl ester (DCF) (12.5 µg/mL; Invitrogen Life Technologies GmbH, Carlsbad, CA, USA) or rhodamin B (20 µg/mL) for 5 min in the dark. It was then centrifuged at 1200× *g* for 10 min at room temperature. Where specified, the platelets were incubated with 50 µg/mL antibodies (e.g., anti-IgG and anti GPIbα) for 30 min and washed (see Table 1). The labeled platelets were suspended in either platelet-poor plasma or modified Tyrode’s buffer (pH 7.4) according to the experimental setup to a final concentration of 150 × 10^3^ platelets/µL, and 200 µL were incubated on collagen-coated coverslips purchased from Neuvitro Corporation (El Monte, CA, USA) for 30 min. The coverslips were washed with PBS (pH 7.4) and the adherent platelets were visualized with the epifluorescence microscope Olympus BX51WI. A total of 4 images were chosen at random per experiment and the percentage of adherent platelet area was calculated on one field of view per image (100 µm × 150 µm).

### 4.3. Flow Cytometric Analysis of Platelet Surface Receptors

Studies were conducted using a BD FACSCanto II flow cytometer with BD FACSDiva software (v6.1.3, BD Biosciences, Heidelberg, Germany) and FlowJo-Software (Tree Star Inc., Ashland, OR, USA). Diluted platelet-rich plasma (PRP) (1:5 with Tyrode’s buffer pH 7.4) was incubated with either 2 or 5 µM ADP (HART Biologicals, Hartlepool, England) for 10 min at room temperature. Activated platelets were stained with PE (phytoerythrin)-labeled JON/A (activated integrin α_IIb_β_3_, Emfret Analytics, Eibelstadt, Germany) for 30 min at room temperature. Samples were supplemented with 400 µL Tyrode’s buffer and analyzed immediately on the flow cytometer, with at least 10,000 events collected per sample. Antibody reactivity is reported as mean fluorescence intensity (MFI). For the analysis of surface receptors, washed platelets were incubated with 3 µg/mL of antibody (Table 1) for 30 min at room temperature or according to manufacturer’s instructions. Samples were supplemented with 400 µL PBS and analyzed immediately on the flow cytometer, with at least 10,000 collected events per sample.

### 4.4. Thromboelastometry

Citrated (3.8%) whole blood was collected by cardiac puncture. A total of 8 µg/mL of bovine type I collagen (Life Technologies Carlsbad, CA, USA) was added to each sample. Blood was recalcified with 20 mM Ca^2+^ directly prior to measurement (50 μL of Ca^2+^-Hepes solution; 100 mM CaCl_2_ + 1 mM Hepes). Clotting time (CT) and clot formation time (CFT) were measured by a whole blood hemostasis analyzer (ROTEM delta, Tem GmbH, Munich, Germany).

## Figures and Tables

**Table 1:** List of antibodies.

Antibody	Clone	Target Molecule	Company
PE-conjugated rat anti-mouse Integrin α_IIb_β_3_	JON/A	activated mouse integrin alpha IIb beta 3	Emfret Analytics, Eibelstad, Germany
Rat anti-mouse GPIbα	5A7	GPIbα	MERU Vasimmune
FITC-labeled Rat-antimouse GPIbβ	Xia.C3	GPIbβ	Emfret Analytics, Eibelstad, Germany
FITC-rat anti-mouse GPVI	JAQ1 Rat IgG2A	GPVI	Emfret Analytics, Eibelstad, Germany
PE-Anti-Mo CD49c	PE-Anti-Mo CD49c	Integrin α2	eBioscience, San Diego, California
FITC-Rat Anti-Mouse CD41	MWReg30	Integrin αIIb	BD Pharmigen San Jose, California
Anti-Rat IgG -FITC	-	-	Emfret Analytics, Eibelstad, Germany
Rat IgG 2aκ	eBR2a	-	eBioscience, San Diego, CA, USA

## Supplementary Materials

The following are available online at https://www.mdpi.com/1422-0067/21/19/7171/s1, Figure S1: Platelet surface receptors.

## Abbreviations

ADP	Adenosine diphosphate
CONV-R	Conventionally raised
CVD	Cardiovascular disease
GF	Germ-free
GP	Glycoprotein
*Ldlr*	Low-density lipoprotein-receptor gene
MAMPs	Microbial-associated molecular patterns
MFI	Mean Fluorescence Intensity
NOD	Nucleotide-binding oligomerization domain
TLRs	Toll-like receptors
TLR2	Toll-like receptor 2
*Tlr2*	Toll-like receptor 2 gene
VWF	Von Willebrand factor
WT	Wild-type
