# Supplementary material for: The Commensal Microbiota Enhances ADP-Triggered Integrin αIIbβ3 Activation and von Willebrand Factor-Mediated Platelet Deposition to Type I Collagen
Source: Int J Mol Sci. 2020 Sep 28;21(19):7171. doi: 10.3390/ijms21197171 (PMC7583822; doi:10.3390/ijms21197171)
Supplement: Supplementary file 1 [file ijms-21-07171-s001.pdf]

Supplementary figure 1

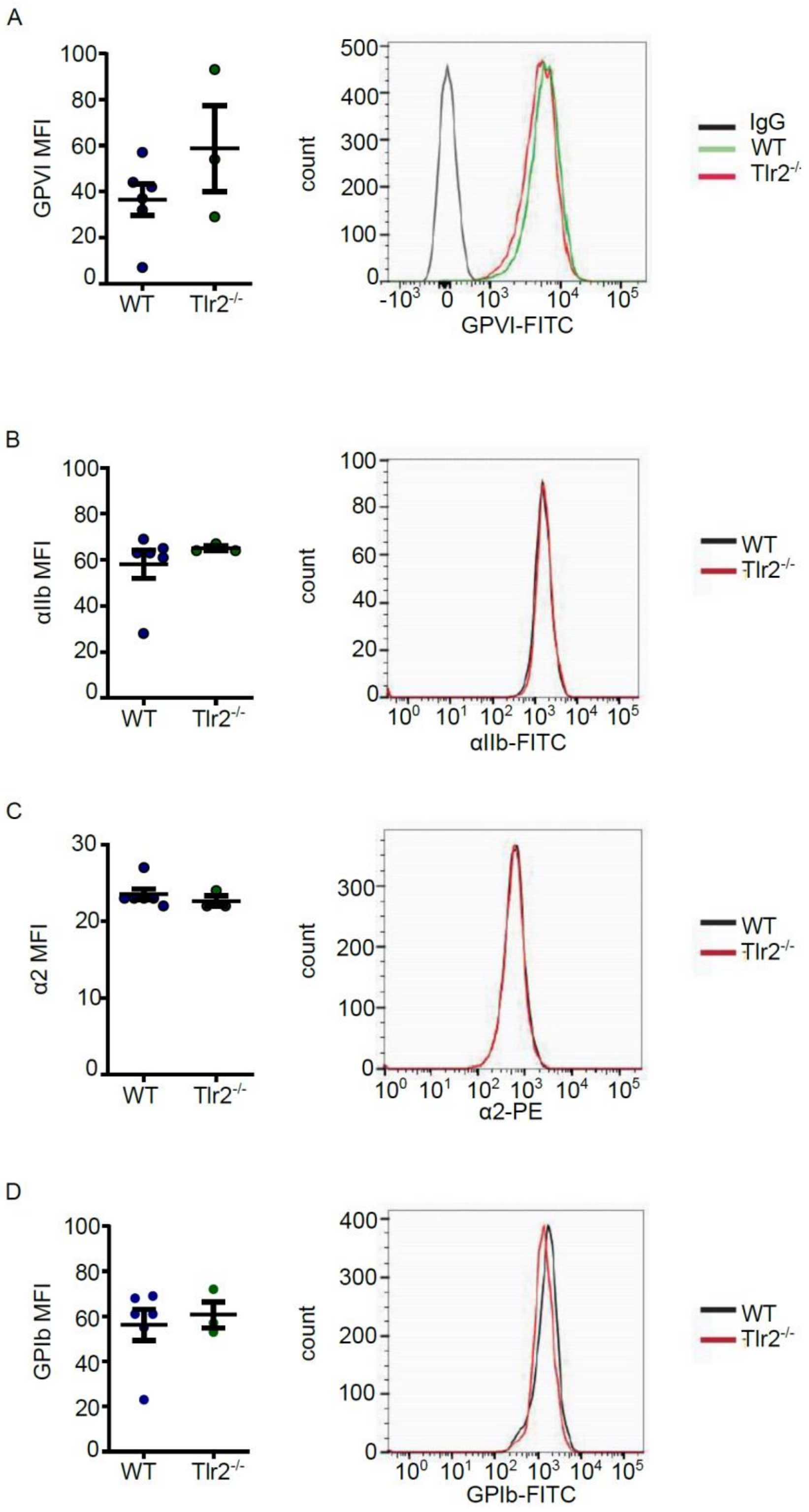

Supplementary Figure 1: Platelet surface receptor mean fluorescence intensity (MFI) and representative histograms of WT (n=5) and $Tlr2^{-/-}$ (n=3) mice quantified by flow cytometry. (**A**) GPVI (**B**) α_{IIb} (**C**) α_2 and (**D**) GPIb. All data were expressed as means ± SEM. Statistical comparisons were performed using the Student's
